# Manipulating high-temperature superconductivity by oxygen doping in Bi_2_Sr_2_CaCu_2_O_8+δ_ thin flakes

**DOI:** 10.1093/nsr/nwac089

**Published:** 2022-05-11

**Authors:** Bin Lei, Donghui Ma, Shihao Liu, Zeliang Sun, Mengzhu Shi, Weizhuang Zhuo, Fanghang Yu, Genda Gu, Zhenyu Wang, Xianhui Chen

**Affiliations:** Key Laboratory of Strongly-Coupled Quantum Matter Physics, Chinese Academy of Sciences, and Department of Physics, University of Science and Technology of China, Hefei 230026, China; CAS Center for Excellence in Quantum Information and Quantum Physics, Hefei 230026, China; Key Laboratory of Strongly-Coupled Quantum Matter Physics, Chinese Academy of Sciences, and Department of Physics, University of Science and Technology of China, Hefei 230026, China; Key Laboratory of Strongly-Coupled Quantum Matter Physics, Chinese Academy of Sciences, and Department of Physics, University of Science and Technology of China, Hefei 230026, China; Key Laboratory of Strongly-Coupled Quantum Matter Physics, Chinese Academy of Sciences, and Department of Physics, University of Science and Technology of China, Hefei 230026, China; Key Laboratory of Strongly-Coupled Quantum Matter Physics, Chinese Academy of Sciences, and Department of Physics, University of Science and Technology of China, Hefei 230026, China; Key Laboratory of Strongly-Coupled Quantum Matter Physics, Chinese Academy of Sciences, and Department of Physics, University of Science and Technology of China, Hefei 230026, China; Key Laboratory of Strongly-Coupled Quantum Matter Physics, Chinese Academy of Sciences, and Department of Physics, University of Science and Technology of China, Hefei 230026, China; Department of Condensed Matter Physics and Materials Science, Brookhaven National Laboratory, Upton, NY 11973-5000, USA; Key Laboratory of Strongly-Coupled Quantum Matter Physics, Chinese Academy of Sciences, and Department of Physics, University of Science and Technology of China, Hefei 230026, China; CAS Center for Excellence in Quantum Information and Quantum Physics, Hefei 230026, China; Key Laboratory of Strongly-Coupled Quantum Matter Physics, Chinese Academy of Sciences, and Department of Physics, University of Science and Technology of China, Hefei 230026, China; CAS Center for Excellence in Quantum Information and Quantum Physics, Hefei 230026, China; CAS Center for Excellence in Superconducting Electronics (CENSE), Shanghai 200050, China; Collaborative Innovation Center of Advanced Microstructures, Nanjing University, Nanjing 210093, China

**Keywords:** high-temperature superconductivity, oxygen doping, electric field effect, all-solid-state field effect transistor, superconductor–insulator transition

## Abstract

Harnessing the fascinating properties of correlated oxides requires precise control of their carrier density. Compared to other methods, oxygen doping provides an effective and more direct way to tune the electronic properties of correlated oxides. Although several approaches, such as thermal annealing and oxygen migration, have been introduced to change the oxygen content, a continuous and reversible solution that can be integrated with modern electronic technology is much in demand. Here, we report a novel ionic field-effect transistor using solid Gd-doped CeO_2_ as the gate dielectric, which shows a remarkable carrier-density-tuning ability via electric-field-controlled oxygen concentration at room temperature. In Bi_2_Sr_2_CaCu_2_O_8+δ_ (Bi-2212) thin flakes, we achieve a reversible superconductor–insulator transition by driving oxygen ions in and out of the samples with electric fields, and map out the phase diagram all the way from the insulating regime to the over-doped superconducting regime by continuously changing the oxygen doping level. Scaling analysis indicates that the reversible superconductor–insulator transition for the Bi-2212 thin flakes follows the theoretical description of a two-dimensional quantum phase transition. Our work provides a route for realizing electric-field control of phase transition in correlated oxides. Moreover, the configuration of this type of transistor makes heterostructure/interface engineering possible, thus having the potential to serve as the next-generation all-solid-state field-effect transistor.

## Teaser

This work reports a novel ionic field-effect transistor (iFET) using solid Gd-doped CeO_2_ as the gate dielectric, which shows remarkable carrier-density-tuning ability by electric-field-controlled oxygen doping at room temperature.

## INTRODUCTION

Correlated oxide materials exhibit rich phenomenology and a wide variety of interesting phases, such as high-temperature superconductivity and colossal magnetoresistance [1]. A generic characteristic of these systems is the close competition of exotic electronic phases that can be traversed by fine-tuning the charge carrier density. An insulating copper oxide, such as La_2_CuO_4_, can be changed into a high-temperature superconductor by partially replacing La with Sr or by increasing the oxygen content to introduce hole doping. In colossal magnetoresistance manganites, the carrier density is the key variable that determines the emergence of ferromagnetic metallic and charge-ordered antiferromagnetic insulating states [2,3]. As the subtle balance of competing interactions in the correlated oxides is sensitive to a small change in carrier density, a continuous fine-tuning method other than chemical substitution is indispensable, from both a fundamental and technological point of view.

In the past decade, pioneering efforts have been made using ionic liquids (ILs) as the dielectric in electric-double-layer transistors (EDLTs) to induce electrostatic charge modulation [4–15]. While the IL-EDLT has been proven to be an effective way of manipulating material properties, there have still been several issues that need to be improved. First, the compatibility of IL-EDLTs with other electronic devices is problematic. The sample is masked by liquid or polymer electrolytes, thus making surface characterization and heterostructure/interface engineering impossible. Second, an electrochemical reaction occurring at the electrolyte-solid interface may change or even damage the sample, especially at room temperature or above. For the high performance and practical application of field-effect transistors (FETs), it is desirable to prepare an all-solid-state FET using solid ionic conductors (SICs) as the dielectric. There have been several successful attempts, based on different types of ions, including proton [16–19], lithium [20–25], sodium [22,26–28] and fluorine [29], to modulate the charge carrier density of materials. Compared with the above-mentioned ions, oxygen is special since it offers a way of tailoring the properties of correlated oxides without introducing additional chemical disorders. Although in a previous report the surface carrier density of an SrTiO_3_ single crystal was regulated due to the accumulation of oxygen ions near the interface [30], the high working temperature and the small tuning depth hinder its further application.

In this work, we have developed a new type of ionic field-effect transistor device based on using a solid oxygen-ionic conductor (OIC), Gd-doped CeO_2_ (GDC), as the gate dielectric. This device, working at room temperature, shows remarkable carrier-modulation ability through the electric-field control of oxygen concentration. To demonstrate the capability of this OIC-based FET device, we have chosen Bi_2_Sr_2_CaCu_2_O_8+δ_ (Bi-2212) as the model system, since its electronic properties are highly sensitive to oxygen stoichiometry. We demonstrate reversible superconductor–insulator transition (SIT) in Bi-2212 thin flakes through driving oxygen ions into and out of the sample by electric field. A phase diagram has been mapped out from the insulating regime all the way to the over-doped superconducting regime, by continuously changing the oxygen content to tune the doping level. Scaling analysis shows that the reversible SIT follows the theoretical description of a two-dimensional quantum phase transition (2D-QPT). Our results establish the OIC-FET as a powerful tool for realizing and studying novel electronic phases in oxide materials.

## RESULTS AND DISCUSSION

**Figure 1.**
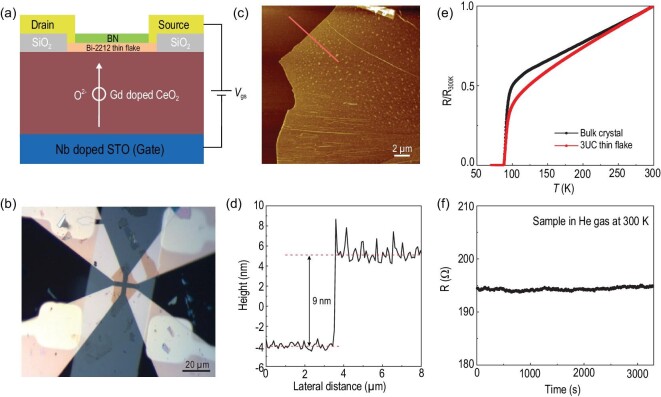
Fabrication and characterizations of 3 UC Bi-2212 devices. (a) Schematic illustration of the device and the gating-induced oxygen intercalation process. (b) Optical image of a typical device with a 3 UC Bi-2212 thin flake. Scale bar, 20 μm. (c) Atomic force microscopy (AFM) image of a 3 UC Bi-2212 thin flake. Scale bar, 2 μm. (d) Cross-sectional profile of the flake along the red line in (c). The thickness of the flake from the step height is determined to be 9 nm, corresponding to 3 UC for Bi-2212. (e) Typical temperature-dependent resistance of a 3 UC Bi-2212 thin flake in comparison with that of the optimally doped single crystal. The data are normalized to the value at *T* = 300 K. (f) Resistance of a 3 UC sample as a function of time in a helium gas atmosphere at 300 K.

Figure 1a depicts a schematic illustration of the OIC-FET device. GDC (Ce_0.8_Gd_0.2_O_1.9_) is chosen as the gate dielectric because of its high oxygen ionic conductivity at relatively low temperatures [30]. The GDC single-crystal films are grown on metallic, (00*l*)-oriented Nb-doped SrTiO_3_ (NSTO) substrates by RF magnetron sputtering, and have smooth surfaces (Fig. S1 of the supplementary information). In this work, we first use Bi-2212 thin flakes with a thickness of 9 nm (3 unit cells (UCs)) as the transport channel. A detailed device preparation procedure is described in the methods section. Figure 1b shows an optical image of a typical Bi-2212 device with a thickness of 3 UCs, and the thickness of the sample is determined by both optical contrast and atomic force microscopy (Fig. 1c and d). It is well known that the exfoliated Bi-2212 thin flakes are extremely sensitive to the environment [31]. In previous reports, monolayer Bi-2212 has been found to be either insulating or superconducting with varying critical temperatures due to the reaction with water vapor and the loss of oxygen dopants [32–34]. In our experiments, the superconducting transition temperature *T*_c_^zero^ (defined as the temperature at which resistance reaches zero, shown in Fig. S2) of the 3 UC Bi-2212 thin flakes ranges from 50 K to 88 K depending on the fabrication process (detailed information can be found in Fig. S3 of the supplementary information). Figure 1e shows the normalized temperature-dependent resistance of a 3 UC Bi-2212 thin flake, in comparison with that of an optimally doped bulk crystal. The thin flake shows a sharp superconducting transition, and the *T*_c_^zero^ (∼88 K) is almost the same as the optimal *T*_c_^zero^ in the bulk. This is consistent with the 2D nature of the superconductivity in Bi-2212 [31]. We have monitored the resistance of the sample stored in a helium atmosphere as a function of time at 300 K. As shown in Fig. 1f, the resistance remains almost unchanged within an hour, indicating that no oxygen loss occurs in the device in a helium atmosphere.

**Figure 2.**
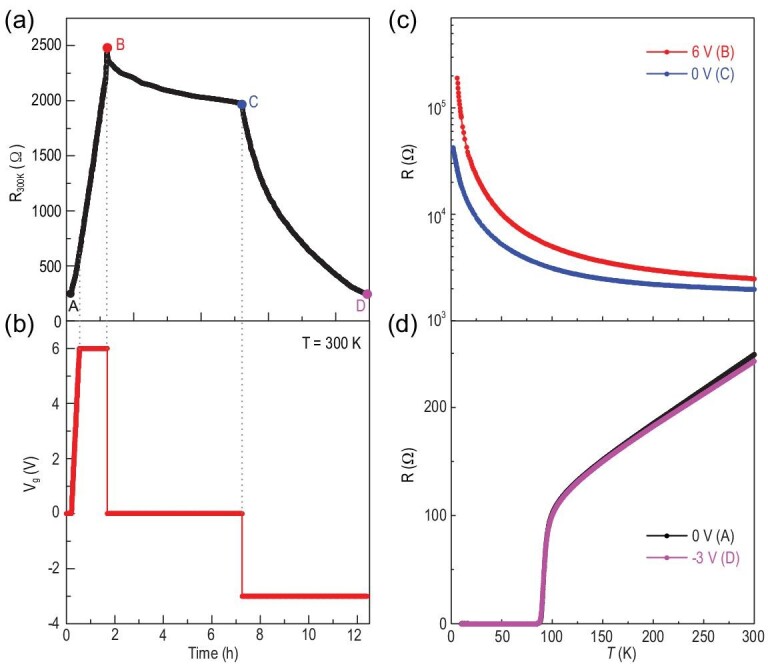
Electric-field-controlled oxygen doping in a 3 UC Bi-2212 thin flake. (a) Time dependence of the resistance for a 3 UC Bi-2212 thin flake at different gate voltages and doping levels. (b) Time-dependent gate voltages applied to the device. (c, d) Temperature dependence of the resistance for the 3 UC Bi-2212 thin flake at different gating voltages in the gating process.

Next, we turn on the gating voltage and test the oxygen-modulation ability of this newly developed OIC-FET device. We first determine the optimal operating temperature for the large gating effect. Below 290 K, the mobility of oxygen ions in the GDC film is quite small, resulting in very weak carrier-modulation capability (Fig. S4 of the supplementary information). On the other hand, above 320 K oxygen in the thin flakes can escape easily even when the flake is protected by hexagonal boron nitride (Fig. S5). These results suggest that a gating temperature of 300 K is best for our OIC-FET devices. Around this temperature, oxygen ions in the GDC film can move under the applied electric field. Oxygen ions can be driven out of the sample under applied positive gate voltages, and vice versa. The loss of oxygen in the Bi-2212 flake under positive gate voltages can lead to a depletion of the hole concentration, resulting in a rapid increase of sample resistance at 300 K. When a gate voltage of 6 V is kept on for ∼70 min, the sample loses oxygen continuously (A–B in Fig. 2a and b). When cooling the device to below 290 K, the carrier concentration can be fixed by freezing the oxygen ions in the GDC. For a given carrier concentration, the temperature-dependent resistance is measured. Figure 2c shows the temperature dependence of resistance at point B, indicating that the sample was driven from the superconducting state (Fig. 2d) to an insulating state by the electric gating. The temperature was swept back to 300 K, then the gate voltage was switched off and kept at zero for ∼6 hours. As shown in Fig. 2a, the resistance of the sample slowly decreases, and then tends to saturate (B to C) at a different value compared to that before gating. This observation provides evidence that the phase transition is not induced by the electrostatic charging at the interface [30] but originates from the altered oxygen concentration in the sample with gating. Then, a gate voltage of −3 V was applied to the device at 300 K, and kept on for ∼5 hours. A negative gate voltage can drive the oxygen ions from the GDC film to go towards the surface of the sample and enter the sample to increase the oxygen concentration of the sample. As a result, the resistance of the sample instantly decreases, and gradually reaches its initial value before gating. Indeed, a superconducting state with *T*_c_^zero^ of 85 K is observed (D), which is almost identical to that measured in the initial state before gating (Fig. 2d). These results suggest that reversible electric-field-controlled oxygen doping dominates the transport properties of Bi-2212 during the gating process in the OIC-FET device.

To further reveal the carrier-modulation capability of OIC-FET devices, we turn to a more systematic study of the phase diagram for the Bi-2212 thin flake. Exfoliated Bi-2212 thin flakes are highly tunable since the cleaved BiO layer is directly exposed to the GDC film, making it easy for oxygen to escape from or enter the thin flake. In order to achieve a fine control of the oxygen doping level, we start with a small gating voltage at 300 K, and slowly increase it after the resistance of the sample saturates. For each gating voltage, the relaxation time ranges from tens of seconds to tens of minutes. Once a target carrier concentration (monitored by the change of resistance at 300 K) is reached, we cool down the device and perform temperature-dependent resistance measurements. A positive gate voltage drives oxygen out of the sample, while a negative one pushes the oxygen into the sample, and these two processes enable us to continuously tune the oxygen doping level and track the evolution of electronic state from a deep under-doped regime all the way to the over-doped regime of Bi-2212, in a single device.

**Figure 3.**
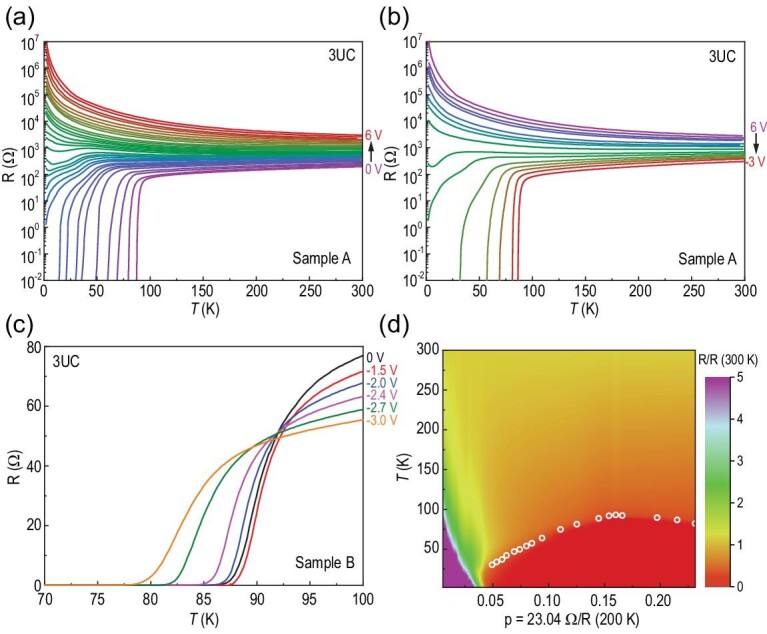
Tunable high-temperature superconductivity by electric-field-controlled oxygen doping in Bi-2212 thin flakes with a thickness of 3 UC. (a) Temperature-dependent resistance for a 3 UC Bi-2212 thin flake (sample A) tuned by electric-field-controlled oxygen doping from the optimally doped regime to the insulating regime. (b) Temperature-dependent resistance of sample A tuned by electric-field-controlled oxygen doping from the insulating regime to the optimally doped regime. (c) Temperature-dependent resistance for another 3 UC Bi-2212 thin flake (sample B) tuned by electric-field-controlled oxygen doping from the near-optimally doped regime to the over-doped regime. (d) The resulting phase diagram as a function of oxygen doping tuned by electric field for a 3 UC Bi-2212 thin flake.

Figure 3a displays the temperature-dependent resistance of a 3 UC Bi-2212 thin flake (sample A) under positive gating voltages from 0 V to 6 V. Initially, the as-exfoliated sample shows high-temperature superconductivity with *T*_c_^onset ^*=^ ^*92.9 K. The positive gate voltage drives oxygen out of the sample, leading to a progressive decrease of the hole doping level, confirmed by Hall coefficient measurements (Fig. S7a). Continuous evolution from a superconducting to insulating state, controlled by increasing the gate voltage, is clearly observed in Fig. 3a. Simultaneously, the room-temperature resistance increases by one order of magnitude from ∼0.2 kΩ to ∼3 kΩ. Interestingly, sweeping the applied gate voltages from 6 V to −3 V can change the sample from an insulating state to a superconducting state reversibly (Fig. 3b), and eventually recover the initial high-temperature superconductivity, with *T*_c_^zero^ ∼86.5 K. To study the tunability of oxygen concentration by electric field in the over-doped regime, a negative gate voltage was applied on another optimally doped Bi-2212 thin flake with a thickness of 3 UC (sample B) and the evolution of superconductivity from a near-optimal-doping to over-doping regime was tracked. Figure 3c shows a zoom-in of the temperature-dependent resistance near the superconducting transition. One can see that the sample achieves optimal doping level, with *T*_c_^zero^ ∼87.5 K for the gating voltage of −1.5 V. With a further increase of the gating voltage to −3 V, the sample is driven to the over-doped regime, and the *T*_c_^zero^ decreases to 77.6 K. It should be mentioned that the electrodes undergo electrochemical corrosion as the gating voltage is further increased, which impedes access to the deeply over-doped regime. It would be of interest to further extend the doping regime for the Bi-2212 thin flake using an OIC-FET device beyond that reported here, by seeking suitable electrodes. A similar transition from an optimally doped superconductor to an insulator has been observed in Bi-2212 thin flakes with thicknesses of 6 UC and 10 UC (shown in Fig. S6).

To determine the evolution of carrier density and establish a detailed phase diagram, we have measured the gate-voltage dependence of Hall resistance *R_xy_* at 200 K. As the sample is tuned from the optimally doped superconductor to an insulator, the Hall coefficient continuously increases, indicating the progressive decrease of the hole carrier density (see Fig. S7a of the supplementary information). In contrast, when the negative gating voltage is increased, the Hall coefficient gradually decreases, suggesting a continuous increase of the hole carrier density, all the way to the over-doped regime (see Fig. S7b of the supplementary information). 1/*R*_H_ as a function of the corresponding inverse resistance (1/*R* (*T* = 200 K)) is summarized in Fig. S8 of the supplementary information, and a nearly linear dependence between 1/*R*_H_ and 1/*R* (*T* = 200 K) is observed. It suggests that the variation of resistance with gating voltage indeed reflects the change of carrier density. In fact, the value of 1/*R* has been frequently used to estimate the doping level for Bi-2212 with different oxygen content [9,13,31,35]. Here, the hole doping level (p) is determined from p = 23.04 Ω/*R* (*T* = 200 K). The value of the constant 23.04 is determined by assuming p = 0.16 at optimal doping [9,13,31]. The normalized resistances as a function of temperature, and the midpoint critical temperatures *T*_c_^mid^ (defined as the temperature at which the resistance drops to half of that at *T*_c_^onset^, shown in Fig. S2) as a function of doping level, are summarized in Fig. 3d. A phase diagram for 3 UC Bi-2212 thin flakes, from the insulating state to the over-doped regime, is mapped out in a single device by modulating the oxygen concentration with an electric field. The resulting phase diagram is quite similar to that of the bulk crystal [36].

**Figure 4.**
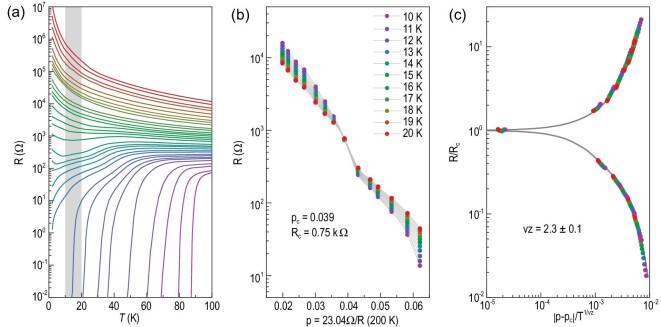
Superconductor-to-insulator transition driven by electric-field-controlled oxygen doping. (a) Temperature-dependent resistance of sample A across the SIT. The gray shaded region indicates the temperature range in which we performed the finite-size scaling analysis. (b) Resistance close to the SIT as a function of doping at fixed temperatures between 10 K and 20 K. Each color refers to a fixed temperature. (c) Scaling analysis of the SIT. The analysis yields a critical exponent of νz = 2.3 ± 0.1.

The SIT observed in the 3 UC Bi-2212 thin flake provides an important example of a continuous quantum phase transition (QPT) driven by oxygen doping. Figure 4a highlights the temperature-dependent resistance for sample A across the SIT. The gray shaded region indicates the temperature range in which we have performed the finite-size scaling analysis. Figure 4b replots the resistance close to the SIT as a function of doping level (p) at fixed temperatures between 10 K and 20 K. Each color refers to a fixed temperature. The critical doping p_c_ is determined to be 0.039, above which the sample shows superconducting transition. The critical resistance (*R*_c_) here is ∼0.75 kΩ. Although it is difficult to precisely calculate the sheet resistance per square in irregular-shaped samples, we can still estimate the value by assuming an aspect ratio of ∼1. The critical sheet resistance per square, normalized by the number of CuO_2_ planes, is found to be ∼9 kΩ, which is comparable to the expected value of (h/(2e)^2^) for the QPT in a purely bosonic model [37]. According to the 2D-QPT theory [37], all resistance data around the SIT should collapse onto a universal scaling function of u = |p − p_c_|/T^1/νz^ on both sides of the SIT. Such scaling does not depend on the exact value of p, and the exponent νz and the critical resistance *R*_c_ encode the essential nature of the SIT. Figure 4c exhibits the resulting R(u) dependence, where the best-fit exponent νz is 2.3 ± 0.1. This value is consistent with the quantum percolation model (νz = 7/3) [38,39]. This νz value is similar to that of ionic-gated YBa_2_Cu_3_O_7-x_ [13] and Pr_2-x_Ce_x_CuO_4_ [15] thin films, but differs from the value observed in La_2−x_Sr_x_CuO_4_ [9], La_2_CuO_4+δ_ [14] and lithium-intercalated Bi_2_Sr_2_CaCu_2_O_8+δ_ thin flakes [35]. The quantum percolation model describes a strongly disordered superconductor [39]. The slight up-turn feature in the resistance at low temperatures implies the existence of granular superconductivity, which makes the scaling analysis difficult below 10 K. The results suggest that the transition from a superconductor to an insulator may involve an intermediate phase. This intermediate phase may be generated by disorder, causing deviation from an ideal 2D-QPT.

## CONCLUSION

In summary, we have developed a novel ionic field-effect transistor based on the use of a solid oxygen-ionic conductor Gd-doped CeO_2_ as the gate dielectric. Working at room temperature, such a transistor shows the remarkable carrier-modulation ability of correlated oxides via electric-field-controlled oxygen doping. The phase diagram of a 3 UC Bi-2212 thin flake is mapped out from the insulating regime all the way to the over-doped regime by continuously changing the oxygen doping level by electric field in a single device. Furthermore, the configuration of this type of transistor allows more systematic characterizations of the sample, e.g. X-ray diffraction to pin down the crystalline structure and surface spectroscopy to reveal the underlying electronic states [40]. Our results represent an important step towards the realization of a long-standing goal to develop complex-oxide-based devices that can be integrated with existing technologies.

## METHODS

### Growth of GDC thin film

The GDC (Ce_0.8_Gd_0.2_O_1.9_) thin film was deposited on the (001)-oriented NSTO substrate by RF magnetron sputtering using a sintered 20% GDC ceramics target with 99.9% purity, prepared by the sol–gel technique. The substrate was treated with oxygen plasma. The RF magnetron sputtering system has a rotating substrate holder for compositional uniformity. The substrate-to-target distance was 90 mm. During the deposition process, the RF power was fixed at 50 W. The total pressure was kept at 1.2 Pa, and the gas flow ratio of argon to oxygen was 3:1. The substrate temperature was maintained at 973 K. In general, the film thickness was controlled between 400 and 800 nm. After deposition, films were cooled in a 20 Pa O_2_ atmosphere.

### Device fabrication

Single crystals of near-optimally doped Bi-2212 (*T*_c_ = 87 K) were grown by the traveling floating zone method. The Bi-2212 thin flakes were mechanically exfoliated from the platelet-like single crystals using scotch tape and PDMS film, and were transferred onto the surface of GDC film with a thickness of ∼700 nm. Before device fabrication, the GDC films were baked on a hot plate at 473 K for an hour to remove possibly adsorbed water. We selected thin flakes with proper shape and thickness using an optical microscope, and characterized the thickness via optical contrast and an atomic force microscope (NX-10, Park system). To avoid chemical pollution and surface contamination from standard lithography techniques, contacts were made by depositing 50 nm of Au through stencil shadow masks previously aligned on the sample in a thermal evaporation system directly connected to the glove box. In order to reduce the leakage current in the process of applying gate voltage, 50 nm of silicon dioxide film was deposited on part of the surface of the GDC film before transferring the sample, to partially isolate the electrodes from the oxygen-ion-conductor film. In order to prevent the loss of oxygen, the sample was completely covered with a piece of exfoliated hexagonal boron nitride with a thickness of ∼15 nm. A back gate electrode was fabricated on the opposite surface of the substrate with silver paste. The whole process of device preparation was performed in a glove box under Ar atmosphere (H_2_O < 0.1 ppm, O_2_ < 0.1 ppm). In addition, the devices were protected by homemade pucks sealed by silicone grease during the transfer process from the glove box to the Quantum Design physical property measurement system (PPMS).

### Transport measurements

Transport measurements were performed using a Quantum Design PPMS. Before loading the device, we had purged the system several times with dry helium gas (99.999%) at 400 K. The longitudinal resistance and Hall resistance were measured using lock-in amplifiers (Stanford Research 830), with the applied current being 500 nA. The gate voltage was supplied by a Keithley 2400 source meter and swept at 300 K. Once the resistance of the sample started to change, the gate voltage was fixed and the sample was then relaxed for tens of seconds or minutes.

## Supplementary Material

nwac089_Supplemental_File
