# Supplementary material for: Manipulating high-temperature superconductivity by oxygen doping in Bi2Sr2CaCu2O8+δ thin flakes
Source: Natl Sci Rev. 2022 May 11;9(10):nwac089. doi: 10.1093/nsr/nwac089 (PMC9671661; doi:10.1093/nsr/nwac089)
Supplement: nwac089_Supplemental_File [file nwac089_supplemental_file.doc]

**Supplementary information**

**Manipulating high-temperature superconductivity by oxygen doping in Bi_2_Sr_2_CaCu_2_O_8+δ_ thin flakes**

Bin Lei^1,2, #,*^, Donghui Ma^1,#^, Shihao Liu^1^, Zeliang Sun^1^, Mengzhu Shi^1^, Weizhuang Zhuo^1^, Fanghang Yu^1^, Genda Gu^3^, Zhenyu Wang^1,2^, and Xianhui Chen^1,2,4,5,*^

1. Key Laboratory of Strongly-coupled Quantum Matter Physics, Chinese Academy of Sciences, and Department of Physics, University of Science and Technology of China, Hefei 230026, China

1. CAS Center for Excellence in Quantum Information and Quantum Physics, Hefei 230026, China
2. Department of Condensed Matter Physics and Materials Science, Brookhaven National Laboratory, Upton, NY 11973-5000, USA
3. CAS Center for Excellence in Superconducting Electronics (CENSE), Shanghai 200050, China
4. Collaborative Innovation Center of Advanced Microstructures, Nanjing University, Nanjing 210093, China

# These authors contributed equally to this work.

* Corresponding author. E-mails: [leibin@ustc.edu.cn](mailto:leibin@ustc.edu.cn) and chenxh@ustc.edu.cn

**
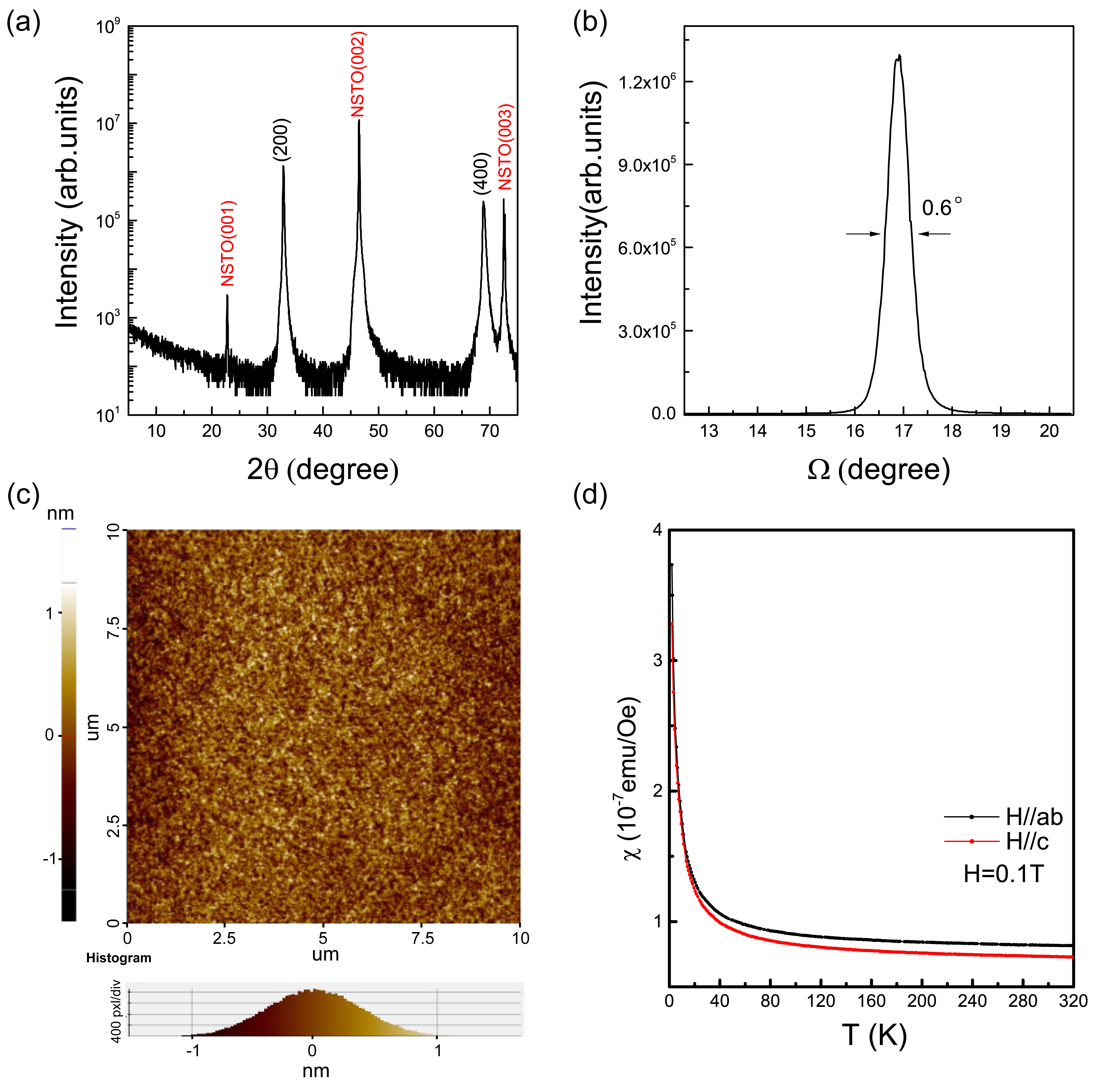
**

**Figure S1.** Characterizations of Gd-doped CeO_2_ film. (a) X-ray diffraction (XRD) patterns of Gd-doped CeO_2_ (GDC) film deposited on (001)-oriented Nb-doped SrTiO_3_ (NSTO) substrate. XRD data was collected by an X-ray diffractometer (SmartLab-9, Rikagu Corp.) with Cu Kα radiation and a fixed graphite monochromator in the 2θ range of 5°–75° at room temperature. The XRD patterns reveal the growth of GDC films along the (200) direction. The Gd-doped CeO_2_ film is single-crystal film, which makes heterostructure/interface engineering convenient. (b) Rocking curve of the (200) reflection of GDC film. The FWHM is 0.6°, indicating the high quality of the film. (c) Typical atomic force microscopy image (10 × 10 *μ*m^2^) of the GDC film with a thickness about 700 nm. The roughness of the surface is ∼ 2 nm. (d) The magnetic susceptibility χ measured at H = 0.1 T as a function of temperature for the Gd-doped CeO_2_ film with a thickness of 700 nm. Data measured with magnetic field parallel to the ab-plane and c-axis are shown in black and red, respectively. The data shows a clear Curie-Weiss behavior without any magnetic transition. It suggests that the Gd-doped CeO_2_ film is nonmagnetic with 20% Gd-substitution of Ce.


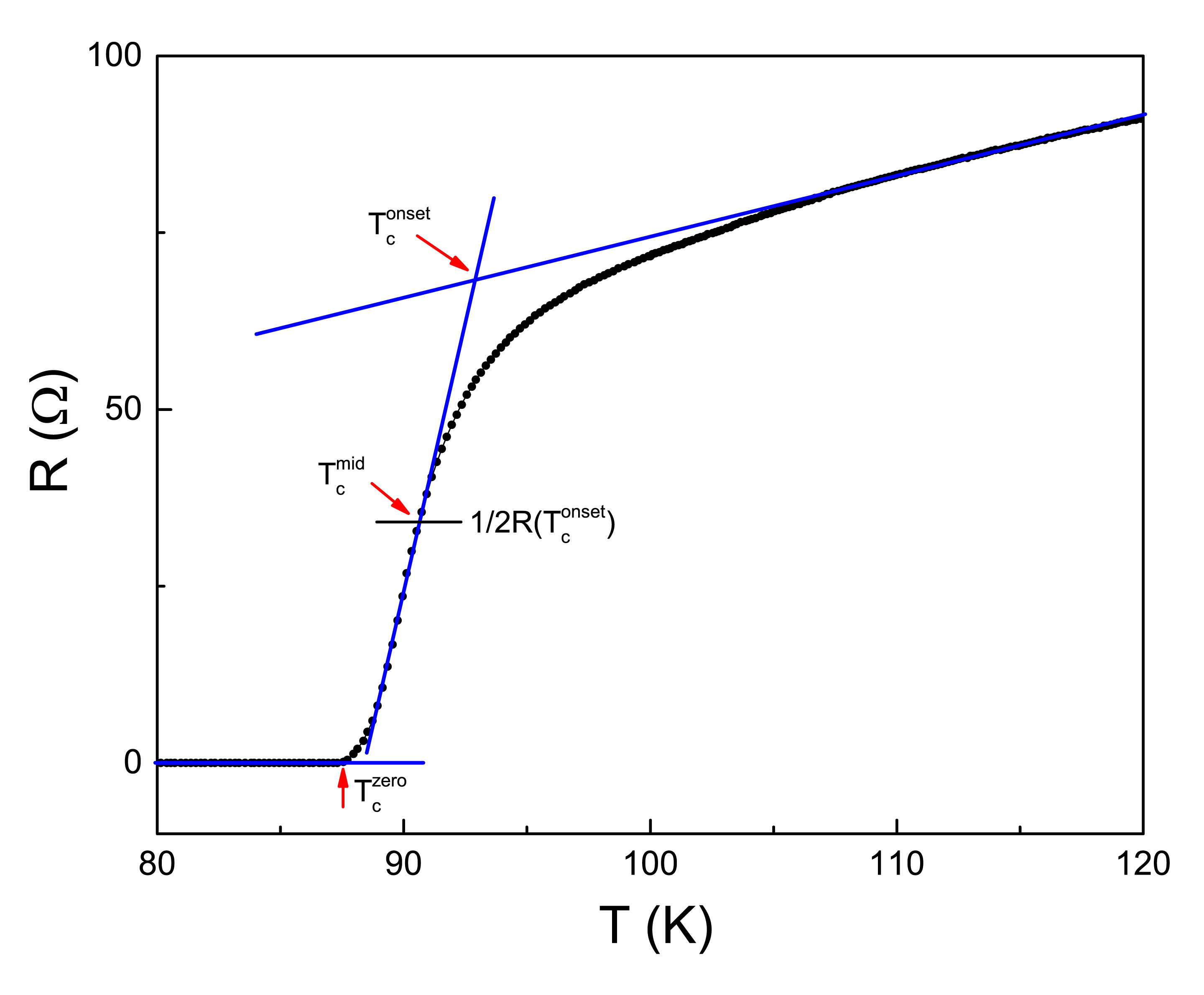


**Figure S2.** Determination of the superconducting transition temperature of Bi-2212. *T*_c_^zero^ is defined as the temperature at which resistance reaches zero. *T*_c_^onset^ is determined from the intersection between the linear extrapolation of the normal state and the superconducting transition. *T*_c_^mid^ is defined as the temperature at which the resistance drops to half of that at *T*_c_^onset^.


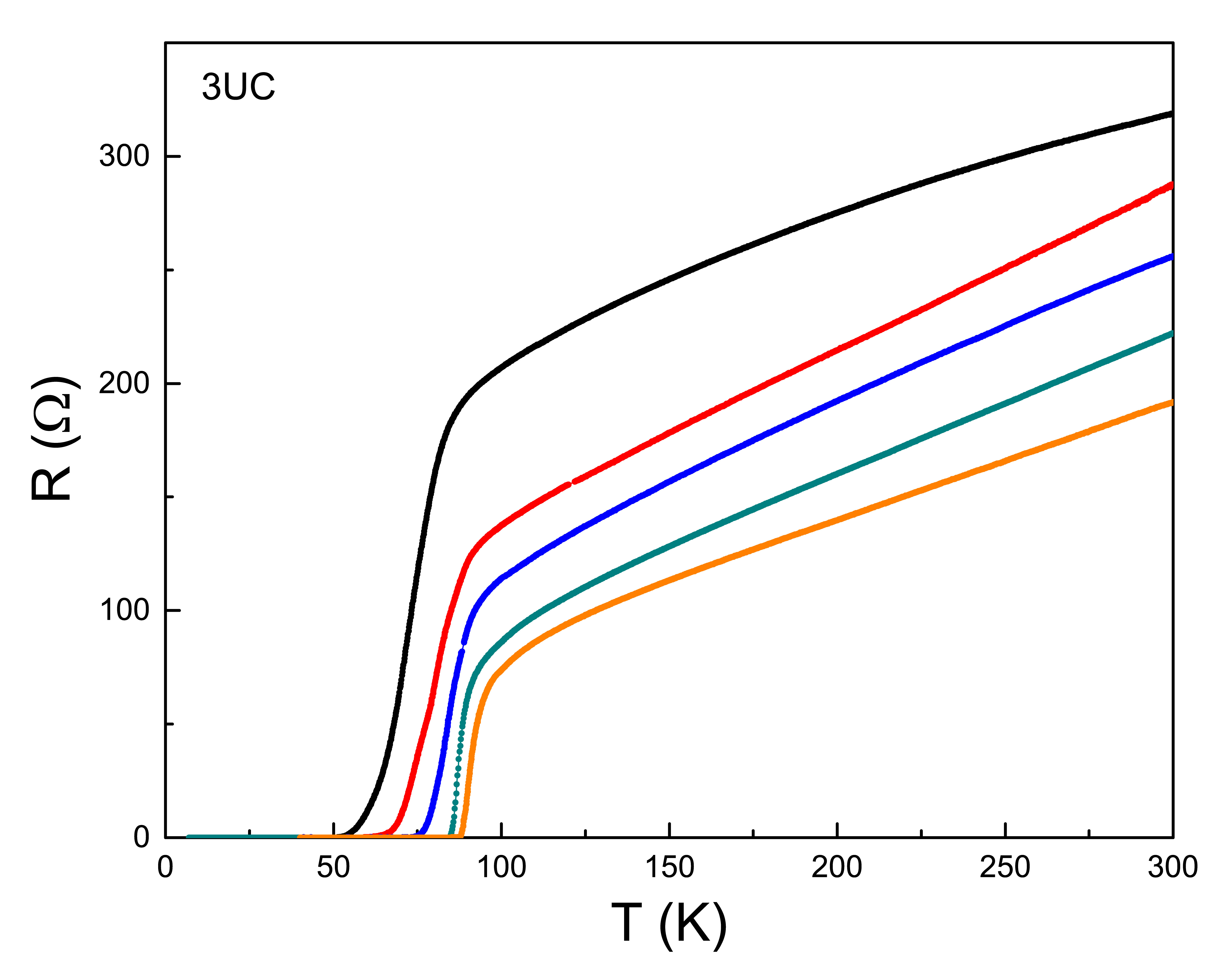


**Figure S3.** The superconducting transition for 3 UC Bi-2212 thin flakes in different fabrication processes. As shown in Figure S3, the superconducting transition temperature *T*_c_^zero^ for the 3 UC Bi-2212 thin flakes ranges from 50 K to 88 K depending on the fabrication processes. First, Bi-2212 thin flake is very sensitive to the photoresist. Standard lithography techniques will cause chemical pollution and surface contamination, resulting in a low *T*_c_^zero^. In our experiments, electrodes were made by depositing 50 nm Au through stencil shadow masks. Second, oxygen is easy to escape from the exfoliated Bi-2212 thin flakes. In order to prevent the loss of oxygen, the sample was completely covered with a piece of exfoliated hexagonal boron nitride. As shown in Fig.1f, the resistance of a 3 UC Bi-2212 thin flake protected with hBN remains almost unchanged within an hour, indicating that no oxygen loss occurs in the device. Third, water in the air can also destroy the superconductivity of the exfoliated Bi-2212 thin flakes. Thus, the whole process of device preparation was operated in the glovebox with Ar atmosphere (H_2_O < 0.1 ppm, O_2_ < 0.1 ppm). Finally, the whole device preparation process should be done as fast as possible. To achieve *T*_c_^zero^ ~ 88 K, the device preparation process should be finished within 2 hours and the device should be measured immediately.


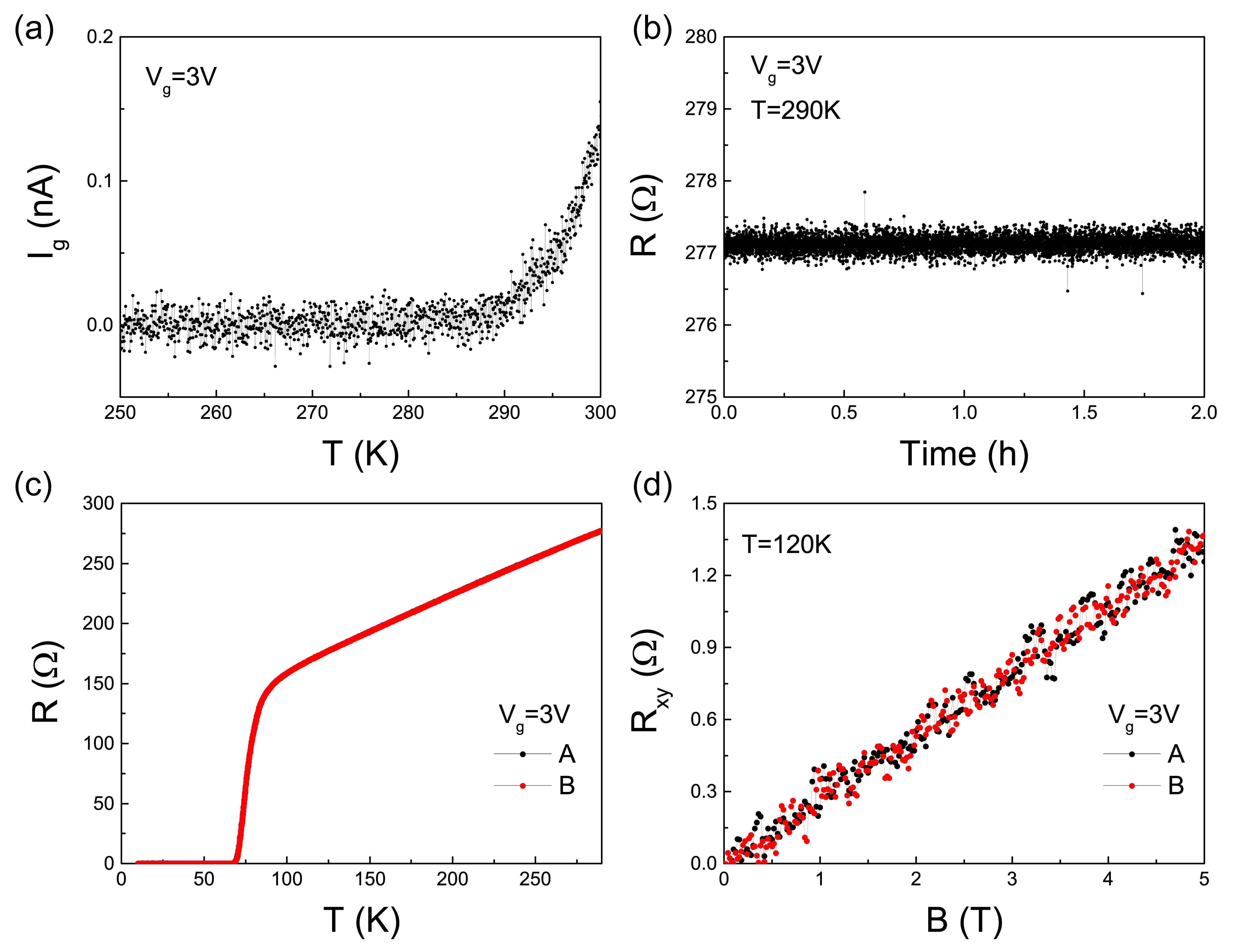


**Figure S4.** (a) Temperature-dependent leakage current (*I*_g_) under an applied gate voltage of 3 V. The leakage current drops to zero below 290 K, suggesting that oxygen ions in the Gd-doped CeO_2_ film are completely frozen. (b) Time-dependent resistance of a 3 UC Bi-2212 flake in a helium gas atmosphere at 290 K, under an applied gate voltage of 3 V. The resistance remains almost unchanged within two hours, indicating that the oxygen concentration is fixed at 290 K. (c) Temperature-dependent resistance of the 3 UC Bi-2212 thin flake before (A) and after (B) a relaxation for two hours at 290 K, under an applied gate voltage of 3 V. The *R*-*T* curves nicely overlap with each other, indicating that the sample state has not changed. (d) Magnetic-field-dependent Hall resistance *R*_xy_ of the 3 UC Bi-2212 thin flake before (A) and after (B) the relaxation for two hours at 290 K under an applied gate voltage of 3 V. The data were measured at 120 K. The curves overlap with each other, indicating that the carrier concentration of the sample does not changes.


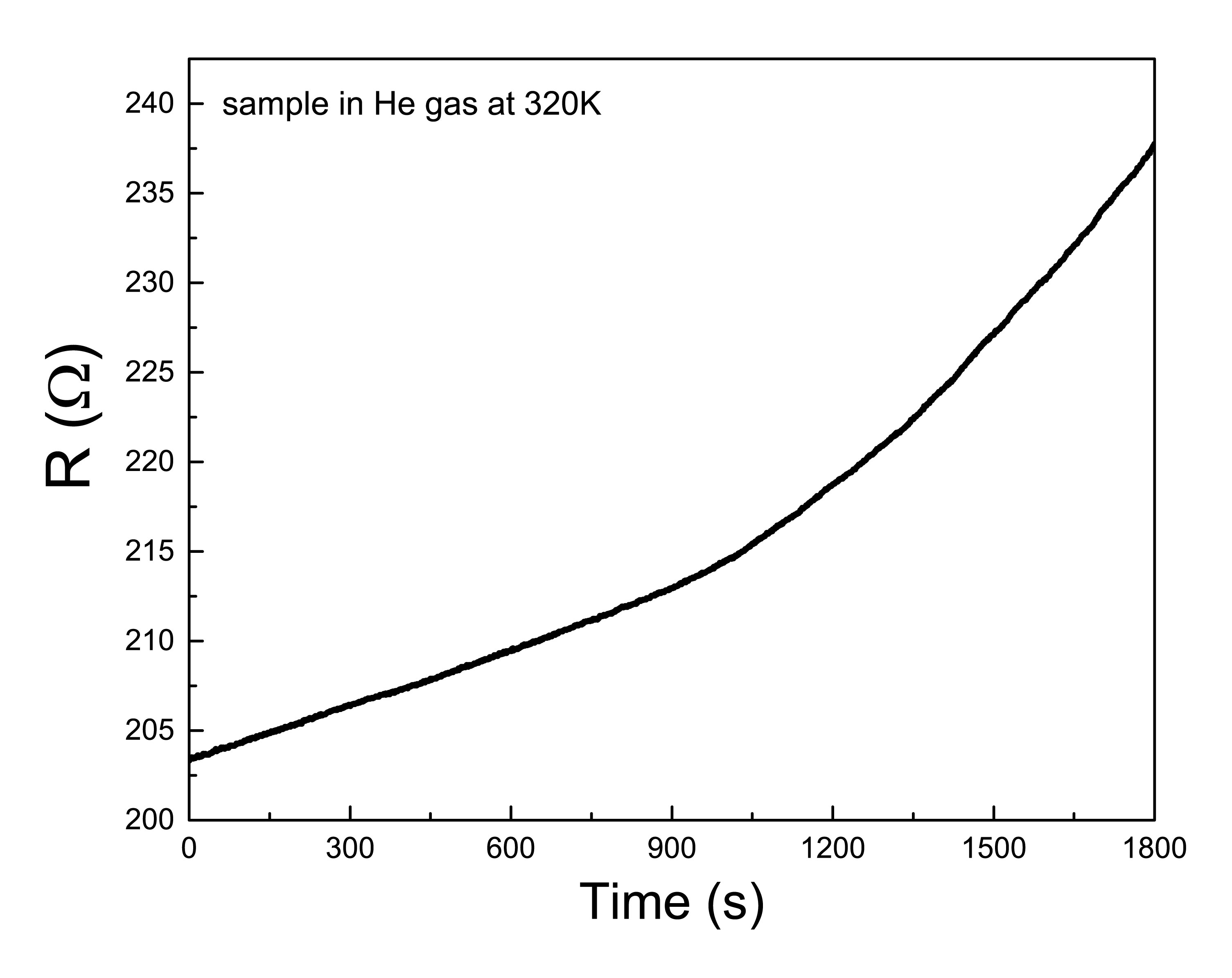


**Figure S5.** Time-dependent resistance of a 3 UC Bi-2212 thin flake kept in a helium gas atmosphere at 320 K. We have monitored the resistance of the Bi-2212 sample stored in a helium atmosphere as a function of time at 320 K. Although the thin flake was protected with hBN, the resistance increases continuously with time, indicating that oxygen loss occurs in the device at 320 K.


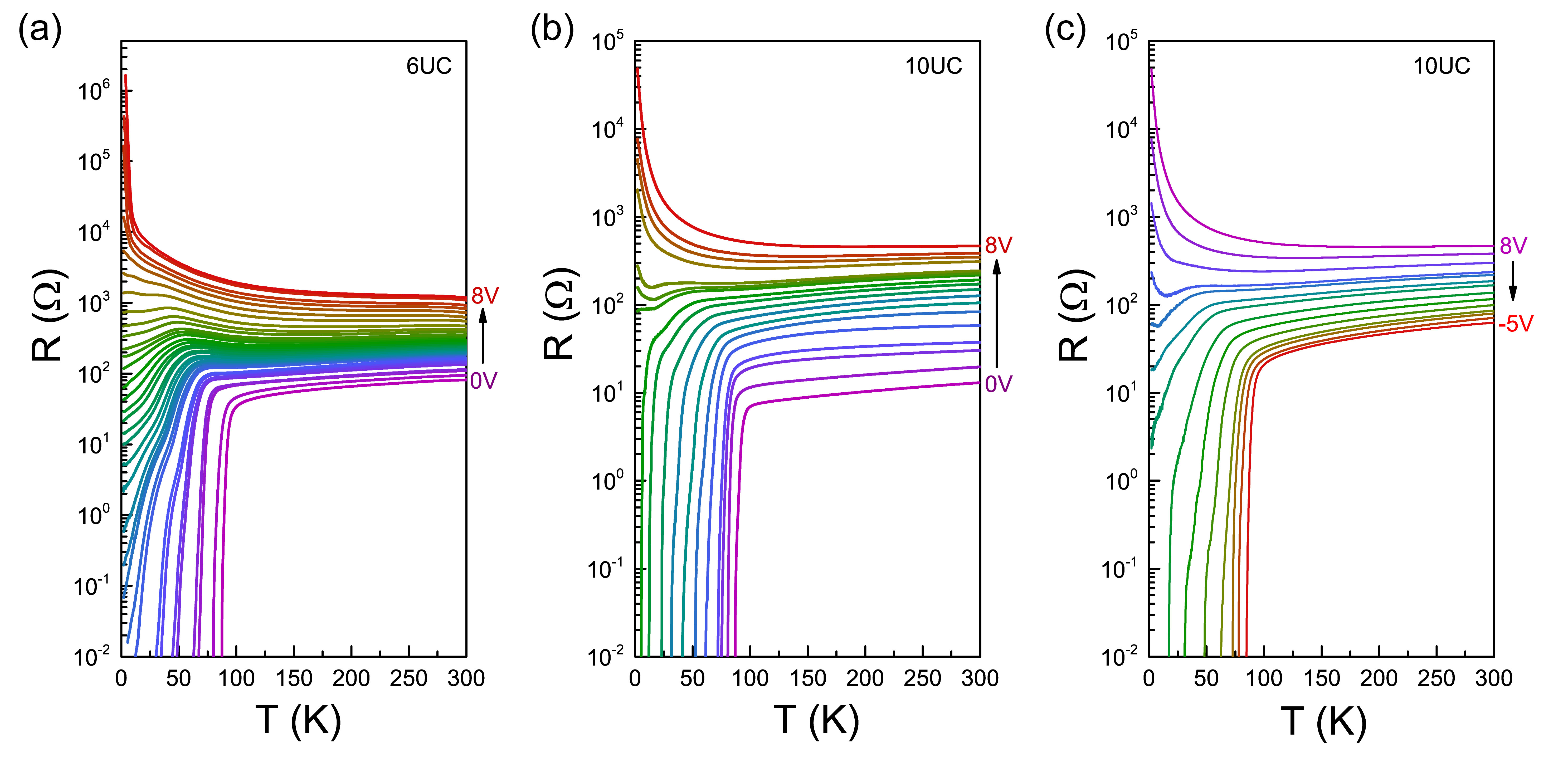


**Figure S6.** SIT for Bi-2212 thin flakes with 6 UC and 10 UC by electric-field-controlled oxygen doping. (a) Temperature-dependent resistance of a 6 UC Bi-2212 thin flake tuned by electric-field-controlled oxygen doping from the optimally-doped regime to the insulating regime. The positive gate voltage was applied from 0 V to 8 V. Initially, the as exfoliated sample shows high-temperature superconductivity with the onset critical temperature *T*_c_^onset^*=* 97 K and reaches zero-resistance at 87.5 K. The positive gate voltage drives oxygen out of the sample, and progressively lowers the hole doping level and induces a transition from superconducting to insulating state. Simultaneously, the room-temperature resistance increases by more than one order of magnitude from about 0.08 kΩ to about 1.2 kΩ. (b) Temperature-dependent resistance of a 10 UC Bi-2212 thin flake tuned by electric-field-controlled oxygen doping from the optimally-doped regime to the insulating regime. The positive gate voltage was applied from 0 V to 8 V. Initially, the as exfoliated sample shows high-temperature superconductivity with the onset critical temperature *T*_c_^onset^*=* 96.2 K and reaches zero-resistance at 86.7 K. The room-temperature resistance increases by more than one order of magnitude from about 0.02 KΩ to about 0.5 KΩ. (c) Temperature-dependent resistance of a 10 UC Bi-2212 thin flake by electric-field-controlled oxygen doping from the insulating regime to the optimally-doped regime. Reversing the applied gate voltages from 8 V to -5 V again recovers the sample back to a superconductor. Eventually, the sample reaches zero-resistance at 84.7 K, which is very close to the initial value.


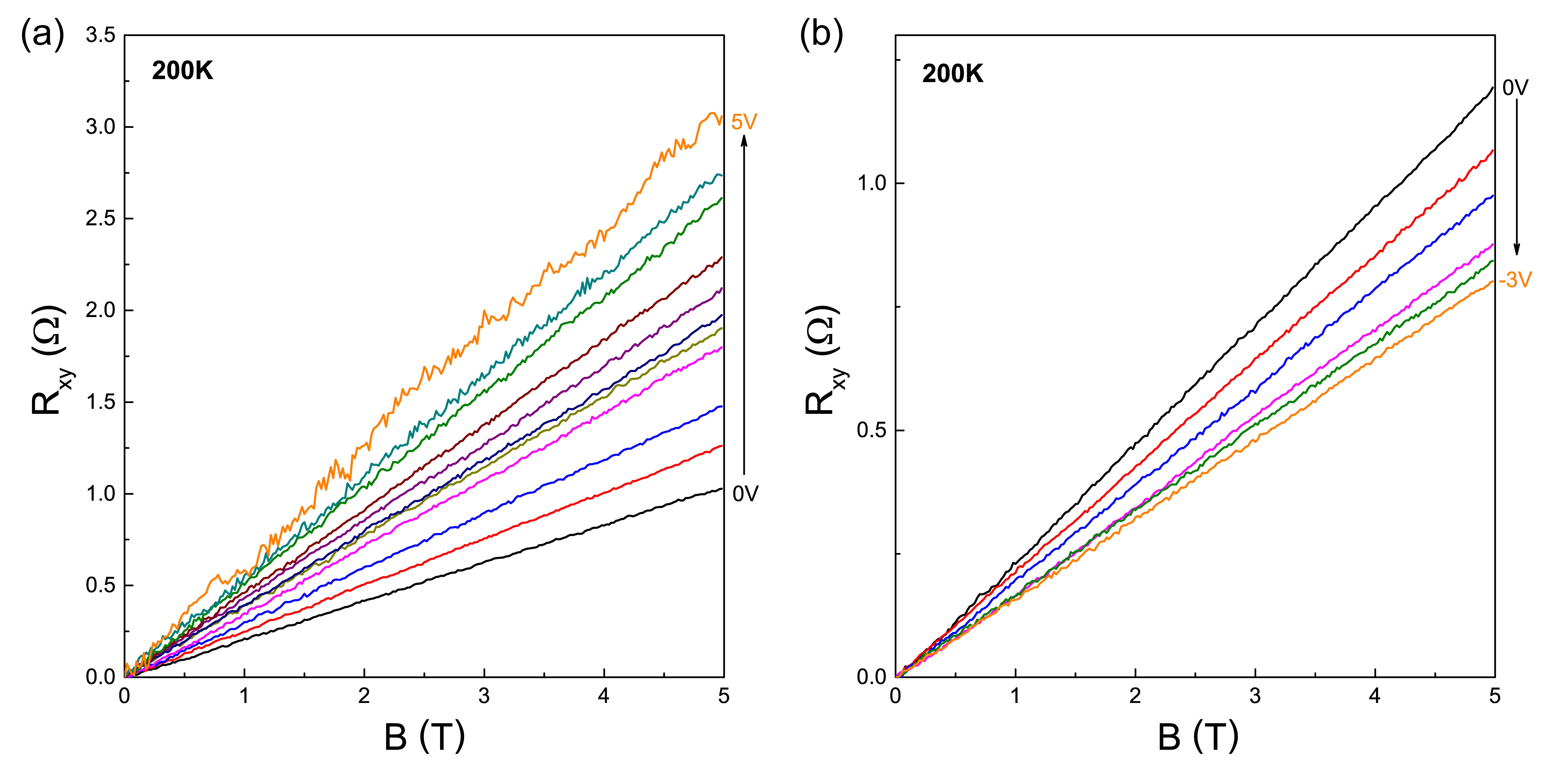


**Figure S7.** Gate-voltage and magnetic-field dependence of the Hall resistance *R_xy_*. (a) Gate-voltage and magnetic field dependence of Hall resistance *R*_xy_ measured at 200 K for sample A. Sample A refers to the same sample in the text of Figure 3(a). The Hall slope increases correspondingly when the sample is tuned from an optimally-doped superconductor to an insulator, indicating the progressive decrease of the hole carrier density. (b) Gate-voltage and magnetic field dependence of Hall resistance *R*_xy_ measured at 200 K for sample B. Sample B refers to the same sample in the text of Figure 3(c). The Hall slope decreases correspondingly when the sample is tuned from a nearly optimal-doped regime to an over-doped regime, indicating the progressive increase of the hole carrier density.


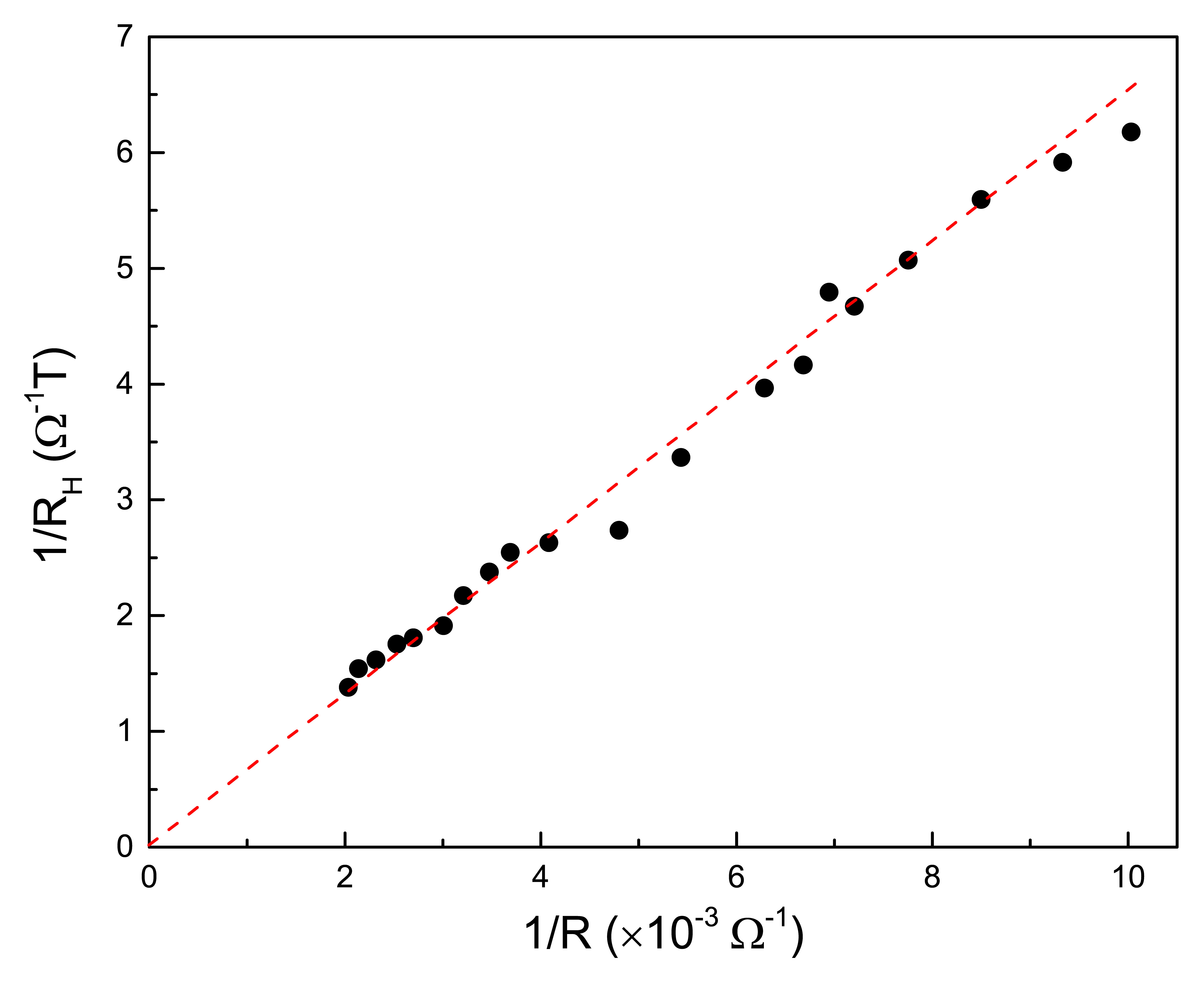


**Figure S8.** Inverse Hall coefficient (1/*R*_H_) as a function of the inverse sheet resistance (1/*R*). We extract *R*_H_ from the magnetic field dependence of Hall resistance *R*_xy_ (shown in Figure S4), and plot 1/*R*_H_ as a function of the corresponding inverse resistance (1/*R*). Both values are obtained at 200 K. A nearly linear trend can be clearly observed. The variation in resistances with gating directly reflects the change of carrier density. The value of 1/*R* (*T* = 200 K) can be adopted to estimate the doping level of sample at different oxygen doping level.


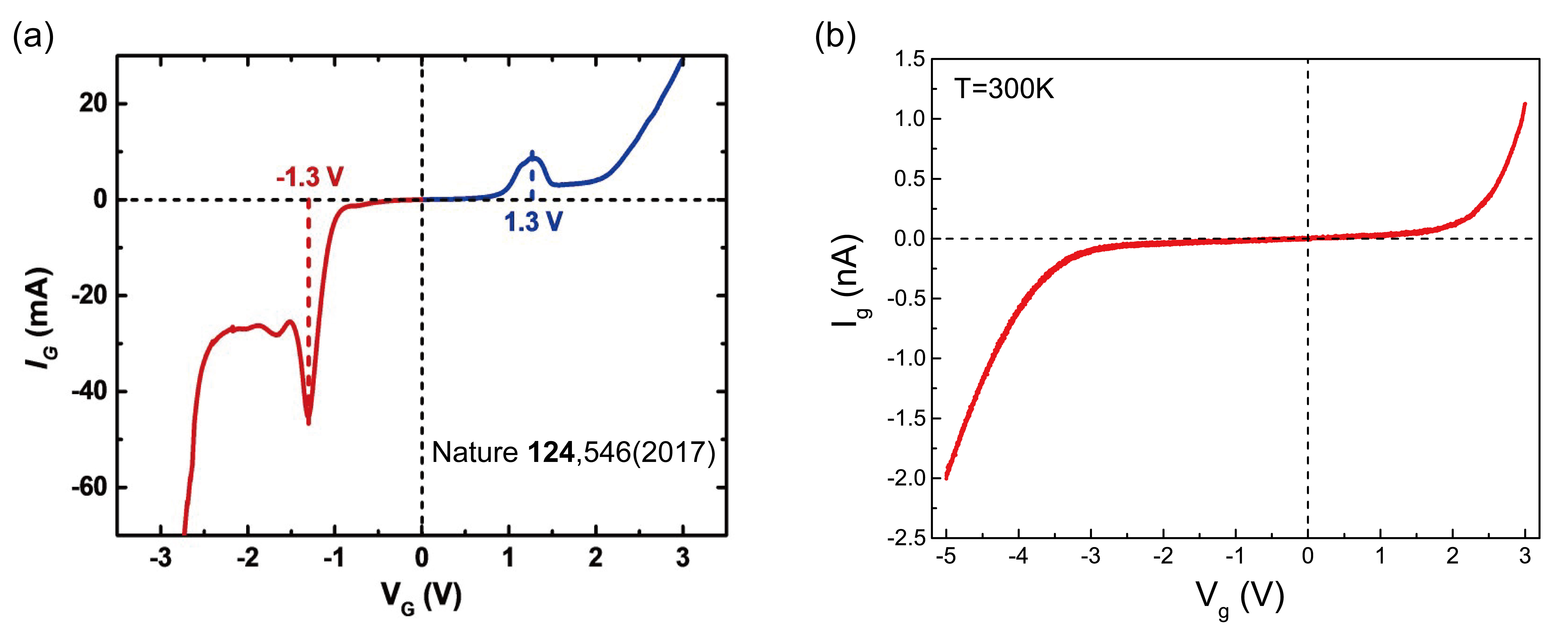


Figure S9. (a) Gating current (*I*_G_) as a function of gating voltage (*V*_G_). This figure is extracted from Nature 124, 546 (2017). The water inside the ionic liquid is decomposed into negatively charged O^2−^ and positively charged H^+^ ions through electrolysis. The curve shows peak features at around 1.3V, being consistent with the standard potential of 1.23 V required for the electrolysis of water. (b) Gating current (*I*_g_) as a function of gating voltage (*V*_g_) in our solid oxygen-ionic-conductor (Gd doped CeO_2_) based field effect transistor device. The curve shows no related feature. This result provides solid evidence that the gating effect in our experiments has nothing to do with the proton intercalation.
